# Birth cohort effect on latent tuberculosis infection prevalence, United States

**DOI:** 10.1186/1471-2334-10-206

**Published:** 2010-07-13

**Authors:** Carla A Winston, Thomas R Navin

**Affiliations:** 1 Division of Tuberculosis Elimination, Centers for Disease Control and Prevention, 1600 Clifton Road NE, Mailstop E-10, Atlanta, Georgia, 30333, USA

## Abstract

**Background:**

Latent tuberculosis infection (LTBI) prevalence in the United States decreased approximately 60% in the three decades between the 1971-1972 and 1999-2000 National Health and Nutrition Examination Survey (NHANES) surveys. We examined the effects of birth cohort on LTBI prevalence over time.

**Methods:**

Using weighted data analysis software to account for NHANES survey design, we calculated the difference in LTBI prevalence between 1971-1972 and 1999-2000 for birth cohorts corresponding to 5-year intervals (1912-1916, 1917-1921,1922-1926, 1927-1931, 1932-1936, 1937-1941, 1942-1946).

**Results:**

LTBI prevalence was significantly lower in 1999-2000 compared to 1971-1972 for cohorts born in 1926 or earlier (19% versus 5%), but not for cohorts born 1927-1946 (9% versus 7%). Adjustment for cohort restriction and foreign-birth did not qualitatively change the results.

**Conclusions:**

Although older age groups have higher rates of TB infection than younger groups, nationally representative U.S. survey data suggest that observed LTBI prevalence in older people represents an underestimate of infection, because of the birth cohort effect and waning immunologic reactivity.

## Background

Two recent publications [1,2] estimate latent tuberculosis infection (LTBI) prevalence in the United States using the 1971-1972 and 1999-2000 National Health and Nutrition Examination Survey (NHANES) data. These studies show that LTBI prevalence has decreased approximately 60% in the three decades between the two surveys. Older age was associated with higher LTBI prevalence in both NHANES time periods; however, neither study examined LTBI in the same birth cohorts over time. Therefore, we compared NHANES 1971-1972 data with NHANES 1999-2000 data to examine the specific effects of birth cohort on LTBI prevalence.

## Methods

Cohorts corresponding to birth years were constructed in 5-year intervals (1912-1916, 1917-1921,1922-1926, 1927-1931, 1932-1936, 1937-1941, 1942-1946) based on available 1971-1972 and 1999-2000 NHANES data by age. Only people age 25-74 years old were offered tuberculin skin testing (TST) in 1971-1972; all individuals 1 year of age and older were tested in 1999-2000. The 1999-2000 data were available only with top-coded age values such that all persons above age 85 were listed as age 85. Thus, we could not report comparisons for people born before 1912, and the comparison for people who in 1971-1972 were age 55-59 years (1912-1916 birth cohort) includes in 1999-2000 all people age 83 or older (born in 1916 or earlier). We report data by birth cohort years and corresponding ages.

Our outcome was LTBI prevalence based on TST positivity, defined as an induration of 10 mm or greater in reaction to purified protein derivative S-1. The protocol for the tuberculosis component of the NHANES was reviewed and approved by the NHANES institutional review board. We analyzed data using SUDAAN 9.0 (RTI International, Research Triangle Park, NC) and SAS 9.1 (SAS Institute, Cary, NC) software to account for NHANES sample survey design and weighting. In brief, we used NHANES Medical Examination Center sample weights to estimate prevalence proportions while accounting for unequal selection and interview and physical examination probabilities. We adjusted for TST nonparticipation according to methods of Bennett et al. [1].

For statistical comparisons, we calculated the difference in prevalence estimates between 1971-1972 and 1999-2000 for each cohort. We used sample variances derived from weighted data to calculate 95% confidence intervals (CI) around prevalence estimates and around prevalence differences. Prevalence differences comparing 1999-2000 with 1971-1972 estimates were considered statistically significant if the 95% CI excluded 0.

## Results

### Latent TB infection prevalence by birth cohort

In 1971-1972 NHANES data, 1012 adults were born between 1912-1946 out of 1492 adults age 25-74 years with a TST result. Out of 7386 persons with a TST result in the 1999-2000 NHANES, 1710 adults were born before 1947.

**Figure 1:**
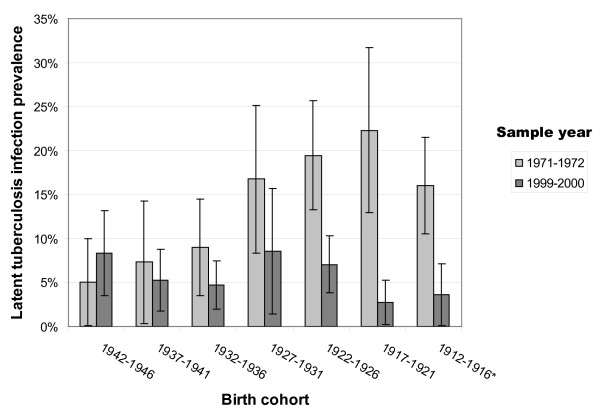
**Latent tuberculosis infection prevalence by birth cohort and sample year**. * National Health and Nutrition Examination Survey 1999-2000 data were top-coded such that all people age 85 and older were identified as 85. Thus the 1912-1916 cohort as analyzed using 1999-2000 data may include persons born in earlier years.

Figure 1 displays LTBI prevalence estimates and associated 95% CI by birth cohort years and NHANES sample year. For cohorts with members born in 1926 or earlier, LTBI prevalence was significantly lower in 1999-2000 compared to 1971-1972. For those born in 1926 and earlier, the overall prevalence of LTBI was 19% in 1971-1972 versus 5% in 1999-2000, representing greater than a three-fold decline. Prevalence differences between NHANES periods were not significantly different for cohorts with members who were 25-44 years old in 1971-1972 (born 1927-1946). For those born 1927-1946, overall LTBI prevalence was 9% in 1971-1972 compared to 7% in 1999-2000. Detailed cohort data are shown in Table 1.

**Table 1:** Latent tuberculosis infection prevalence by birth cohort and sample year

Birth cohort	Age in years in 1971-1972	Age in years in 1999-2000	Latent tuberculosis infection prevalence 1971-1972	Latent tuberculosis infection prevalence 1999-2000	Prevalence difference	95% Confidence interval (CI) around difference	
1942-1946	25-29	53-57	5.05%*	8.34%	3.29	-3.29	9.87
	(no. = 129)	(no. = 245)					
1937-1941	30-34	58-62	7.30%*	5.28%*	-2.01	-9.64	5.61
	(no. = 123)	(no. = 293)					
1932-1936	35-39	63-67	8.97%*	4.70%	-4.26	-10.23	1.70
	(no. = 130)	(no. = 321)					
1927-1931	40-44	68-72	16.78%	8.51%*	-8.27	-18.84	2.31
	(no. = 110)	(no. = 270)					
1922-1926	45-49	73-77	19.47%	7.06%	-12.41	-19.25	-5.57
	(no. = 179)	(no. = 217)					
1917-1921	50-54	78-82	22.29%	2.71%*	-19.58	-29.18	-9.98
	(no. = 191)	(no. = 214)					
1912-1916^†^	55-59	83-85+	16.05%	3.60%*	-12.46	-18.79	-6.12
	(no. = 150)	(no. = 150)					

Selection bias analysis comparing ages available in 1971-1972 vs. 1999-2000							

1912-1946^†^	TOTAL of cohorts	TOTAL of cohorts	13.47%	6.25%	-7.22	-3.31	-11.12
	25-59	53-85+					
	(no. = 1012)	(no. = 1710)					

Not applicable	TOTAL population	TOTAL population	14.25%	5.70%	-8.55	-5.067	-12.04
	25-74	25-74					
	(no. = 1492)	(no. = 3012)					

Not applicable	TOTAL population tested	TOTAL population tested	14.25%	4.18%	-10.07	-6.68	-13.46
	25-74	1-85+					
	(no. = 1492)	(no. = 7386)					

* Estimates are unstable because there are too few individuals represented in these subgroups.

† National Health and Nutrition Examination Survey 1999-2000 data were top-coded such that all people age 85 and older were identified as 85. Thus the 1912-1916 cohort as analyzed using 1999-2000 data may include persons born in earlier years.

### Assessment of prevalence bias

To assess potential bias due to cohort restriction, we compared LTBI prevalence of the overall NHANES populations tested to persons eligible for our analysis. Including older persons (born before 1912) in the 1971-1972 population yielded nominally higher LTBI estimates than our cohort eligible population, while including younger persons (born after 1946) in 1999-2000 resulted in slightly lower LTBI estimates than our cohort analysis (Table 1).

**Figure 2:**
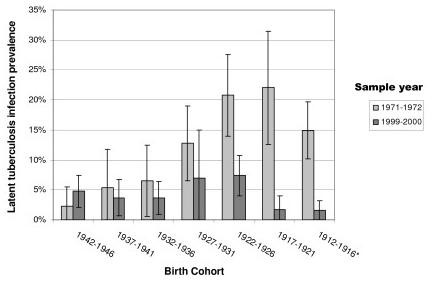
**Latent tuberculosis infection prevalence by birth cohort and sample year, U.S.-born individuals**. * National Health and Nutrition Examination Survey 1999-2000 data were top-coded such that all people age 85 and older were identified as 85. Thus the 1912-1916 cohort as analyzed using 1999-2000 data may include persons born in earlier years.

To account for potential confounding by country of origin, we repeated the analyses among 946 of 1012 (93%) persons in 1971-1972 and 1336 of 1710 (78%) persons in 1999-2000 who were U.S.-born. Results were within 2-3 percentage points of LTBI prevalence difference estimates for the combined U.S. and foreign-born population, with the equivalent conclusion: LTBI prevalence in 1999-2000 was significantly lower than in 1971-1972, achieving statistical significance among cohorts born in 1926 or earlier (Figure 2). Data for foreign-born individuals were too sparse for meaningful stratified analysis: five of seven foreign-born cohorts in 1999-2000 contained fewer than five individuals who were LTBI positive; all 95% confidence intervals were greater than 50 percentage points wide.

## Discussion

When comparing 1971-1972 with 1999-2000 NHANES data, we found a significant decline in LTBI prevalence among people born before 1926 but not for younger birth cohorts. Differences in LTBI prevalence in a birth cohort over time are a balance between new infections and waning of immunologic response to previous infections [3,4]. Our data suggest that for older birth cohorts, the balance is in favor of waning response to previous infections. For younger birth cohorts, the acquisition of new infections may tip the balance in the other direction.

Loss of immunologic reactivity to *Mycobacterium tuberculosis *in humans can be demonstrated by "two-step testing" with purified protein derivative (PPD), such as during repeated screening of healthcare workers. People who test negative in response to the first PPD test and subsequently test positive after a second test within a short period of time (~ 2 weeks) are thought to represent immunologic boosting of a previous infection [3]. Some studies have reported that PPD boosting is associated with age. Among hospital workers in Saudi Arabia, immunologic boosting was more common among workers over the age of 45 than younger workers [5]. Similarly, boosting was more than three times as common in employees 45 years and older than younger employees in a study of 10 hospitals throughout the United States [6]. In contrast, a study in young (mean 21.4 years) healthcare workers in Montreal did not find an association between boosting and age [7]. Perhaps waning reactivity to PPD is not observable before a certain age, such as 45 years.

A potential limitation of our analysis is that LTBI prevalence estimates were based on reaction to PPD S-1, which could include false-positive results based on reactivity to nontuberculous mycobacteria (NTM) or bacillus Calmette-Guérin (BCG) vaccination [8,9], An alternate definition of LTBI incorporating results for PPD-Battey in addition to PPD S-1 to assess NTM cross-reactivity yielded no significant differences in prevalence estimates compared with LTBI based on PPD S-1 alone [1]. Furthermore, the 1999-2000 NHANES reported a higher prevalence of NTM sensitization compared with the 1971-1972 NHANES, and this difference was significant among foreign-born persons [10]. Increases over time in reactivity to NTM would increase LTBI prevalence estimates, rather than contribute to decreases in LTBI that we report. In addition, the waning effect of BCG vaccination is not likely to bias our findings. Prevalence estimates considering foreign-born individuals with a BCG scar as not having LTBI did not differ from estimates ignoring BCG scars [1]; moreover, the NHANES foreign-born population was larger in 1999-2000 than 1971-1972, thus, potential misclassification due to BCG would result in higher, rather than lower, LTBI estimates in 1999-2000. Nonetheless, future research on LTBI will benefit from newer diagnostic technologies with higher specificity for *M. tuberculosis*, such as interferon gamma release assays[11]. We did not analyze data separately for persons who had ever been prescribed treatment for TB or LTBI since this group represented only 1.3% of the total population in 1999-2000 NHANES [1,2].

The reduced LTBI prevalence among earlier birth cohorts that we observed could be influenced by selective mortality, to the degree to which people with LTBI progress to TB disease and die from TB or comorbid conditions. Since LTBI is more prevalent among vulnerable populations [1], people with high rates of LTBI in earlier birth cohorts may experience higher mortality, resulting in lower LTBI rates among survivors. We cannot directly evaluate the influence of self-cure of TB, waning immunity, or selective mortality, since we compared birth cohorts across NHANES sample years, rather than longitudinal follow-up of the same individuals over time. Another limitation was small sample size in some cells, which restricted our ability to stratify analyses. Finally, data were not available for a detailed analysis of people born before 1912 or after 1946. However, comparison of LTBI prevalence -- a relatively rare outcome -- is strengthened by our use of large, nationally representative samples with consistent TST protocols. NHANES household sampling mitigates potential bias, in that the most vulnerable populations for LTBI (homeless or incarcerated people, and residents of long-term care facilities) are excluded. Importantly, our findings did not appear to be driven by greater inclusion of foreign-born people in 1999-2000 NHANES, as results were similar when restricted to the U.S.-born.

## Conclusions

Our study is the first that we are aware of to describe the change in LTBI prevalence associated with age in relationship to the effects of birth cohort, as previously described with respect to TB disease [12]. Although previous studies have shown that older age groups have higher rates of TB infection than younger groups, our nationally representative data suggest that observed LTBI prevalence in older people represents an underestimate of infection, because of the birth cohort effect and waning immunologic reactivity.

## Competing interests

The authors declare that they have no competing interests.

## Authors' contributions

CAW had access to the study data, performed statistical analyses, and drafted the manuscript. TRN conceived of the study and helped to draft the manuscript. Both authors read and approved the final manuscript.

## Pre-publication history

The pre-publication history for this paper can be accessed here:

http://www.biomedcentral.com/1471-2334/10/206/prepub
